# Syndecan-1 (CD138), Carcinomas and EMT

**DOI:** 10.3390/ijms22084227

**Published:** 2021-04-19

**Authors:** John R. Couchman

**Affiliations:** Biotech Research & Innovation Centre, University of Copenhagen, 2200 Copenhagen, Denmark; john.couchman@bric.ku.dk

**Keywords:** proteoglycan, tumor, heparan sulfate, glycosaminoglycan, cadherin

## Abstract

Cell surface proteoglycans are known to be important regulators of many aspects of cell behavior. The principal family of transmembrane proteoglycans is the syndecans, of which there are four in mammals. Syndecan-1 is mostly restricted to epithelia, and bears heparan sulfate chains that are capable of interacting with a large array of polypeptides, including extracellular matrix components and potent mediators of proliferation, adhesion and migration. For this reason, it has been studied extensively with respect to carcinomas and tumor progression. Frequently, but not always, syndecan-1 levels decrease as tumor grade, stage and invasiveness and dedifferentiation increase. This parallels experiments that show depletion of syndecan-1 can be accompanied by loss of cadherin-mediated adhesion. However, in some tumors, levels of syndecan-1 increase, but the characterization of its distribution is relevant. There can be loss of membrane staining, but acquisition of cytoplasmic and/or nuclear staining that is abnormal. Moreover, the appearance of syndecan-1 in the tumor stroma, either associated with its cellular component or the collagenous matrix, is nearly always a sign of poor prognosis. Given its relevance to myeloma progression, syndecan-1-directed antibody—toxin conjugates are being tested in clinical and preclinical trials, and may have future relevance to some carcinomas.

## 1. Introduction

**Figure 1:**
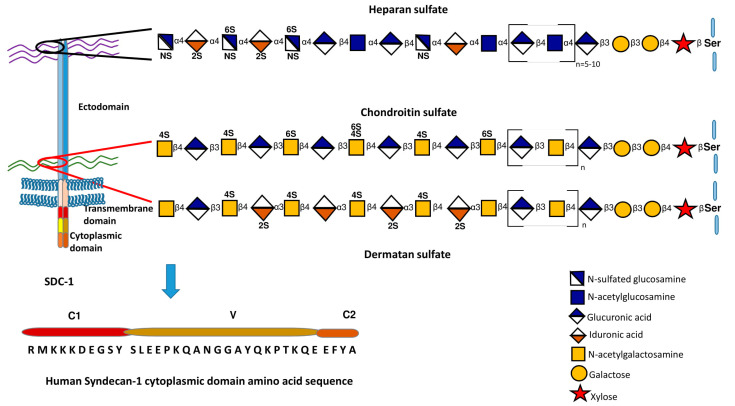
A schematic representation of syndecan-1 structure, cytoplasmic domain sequence and approximate location of heparan and chondroitin/dermatan sulfate chains. The synthesis of these glycosaminoglycans is also summarized. Further details of glycosaminoglycan synthesis and its regulation can be found in [9,10,11].

Syndecan-1 (CD138) is the founder member of a small family of transmembrane proteoglycans. It was first characterized biochemically in the NMuMg murine mammary epithelial cell line, before being cDNA cloned from the same source [1,2,3]. In short order, three further mammalian members of the family were successfully cloned, though none has the same distribution as syndecan-1 [4]. Through immunohistochemistry, it became clear that syndecan-1 had an epithelial expression pattern in particular, though it is present in other tissue types, notably plasma cells of the B lineage and some stromal cells [5,6]. Wherever it has been analyzed, syndecan-1 bears heparan sulfate chains, but in some epithelia, there are additional chondroitin or dermatan sulfate chains. This was noted particularly in non-stratifying epithelial cell types, including the NMuMg line [7,8]. It is proposed that the chondroitin/dermatan sulfate chains are located more membrane-proximally, while the heparan sulfate chains are located distally [8]. The synthesis of glycosaminoglycans has been covered extensively [9,10,11] (see also Figure 1). From the biological and biochemical point of view, heparan sulfate is one of the most variable and anionic of polysaccharides. The net charge results from extensive sulfate and carboxylate groups but the extent of sulfation and its location along the chains are subject to considerable variation. It may be that in a defined location, e.g., the hepatocyte, the variation is less and it is known that HS chains from this source are of higher net charge than other locations [12]. This can impact the affinity and specificity of ligands that bind to the chains.

The literature is replete with reports of heparin (or heparan sulfate)-binding polypeptides, and it is now clear that scores of potential ligands for heparan sulfate include examples of extracellular matrix components, growth factors, cytokines, chemokines, enzymes, lipid metabolites and morphogens [10,13,14]. Very recently, a comprehensive analysis of the glycosaminoglycan interactome has been published [15]. In addition, syndecan-1 core protein can interact directly with integrin receptors, which in concert with growth factor receptors, can lead to complex formation that influences a number of properties revolving around the actin cytoskeleton, such as adhesion and migration [16,17]. The use of peptides corresponding to the binding site on the syndecan core protein as competitors led to the term synstatins, i.e., inhibitors of syndecan-1 function [18,19].

All syndecans have a single transmembrane domain that includes a gly-x-x-x-gly motif that is a strong promoter of dimer formation [20,21,22]. Likely, syndecans are dimers in their native state [23]. The cytoplasmic domain of syndecan-1 has remained an enigma. Many years ago we assigned syndecan cytoplasmic domains into three regions, C1, V and C2. The membrane proximal C1 and distal C2 (Figure 1) are highly conserved and can be recognized across species and phyla, for example in Caenorhabditis elegans and Drosophila [23,24]. Interacting partners for the C1 and C2 regions have been elucidated. The former binds actin-associated proteins and is potentially involved in endocytosis [25]. The C2 region interacts with a number of PDZ proteins, syntenin being the first reported [26] that was later associated with the formation of exosomes [27,28], but also important in cytoplasmic trafficking [29,30]. The central V (variable) region is distinct to each syndecan, though in the case of mammalian syndecan-1, its sequence is very similar to its nearest relative, syndecan-3. However, binding partners for the syndecan-1 V region have remained elusive. Data from the equivalent region of syndecan-4 suggest that syndecan-specific signaling emanates from interactions through the V region [6], so this remains an important gap in our understanding. In total, little is understood of the signaling repertoire of this proteoglycan.

## 2. Syndecan-1 and the Epithelial Phenotype

**Figure 2:**
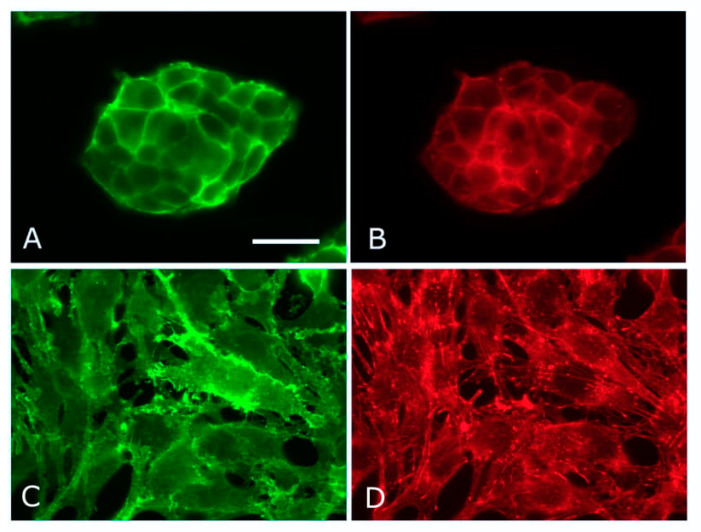
Epithelial–mesenchymal transformation in NMuMg cells. Control (**A**,**B**) and syndecan-1-negative (**C**,**D**) NMuMg cells stained for β–catenin (**A**,**C**) and F-actin (**B**,**D**). Syndecan-1 was depleted by CRISPR/Cas9 technique. The loss of epithelial morphology accompanies syndecan-1 depletion, and the resulting fibroblastic morphology is accompanied by microfilament bundle formation. Scale bar = 50 µm.

Not long after the initial characterization of syndecan-1 from NMuMg cells, a key experiment was performed. Depletion of the core protein by antisense RNA led to a profound change in cellular phenotype [31]. A similar experiment is shown in Figure 2. Formerly epithelial cells became mesenchymal and it was subsequently established that loss of the syndecan was accompanied by depletion of E-cadherin from the cell surface [31]. This provided a valuable insight and suggested that syndecan-1 was in some way essential in maintaining epithelial morphology, and by extension, the formation of E-cadherin containing adherens junctions. Moreover, experiments with transformed mammary epithelial cells showed reciprocity in the syndecan-1-E-cadherin relationship. Manipulation of E-cadherin levels had a corresponding impact on levels of cell surface syndecan-1 [32]. The molecular basis of these data remains unknown, but was shown to be post-transcriptional, i.e., mRNA levels were unchanging. This suggests impact on subcellular localizations and trafficking. Syndecan-1 itself has not been reported as an adherens junction component and neither has a direct interaction between the proteoglycan and the cadherin. Moreover, as has been shown several times, the syndecan-1 null mouse is viable, fertile and does not display severe developmental defects while epithelial morphology, in particular, seems unaffected. Epithelial repair processes in the postnatal mouse are compromised, however [33]. It is also worth bearing in mind that the syndecan-1 gene has been lost in bony fishes, so they express only three syndecans [34].

One potential explanation for the lack of a developmental phenotype in epithelia may be redundancy. There are four mammalian syndecans, and syndecan-4, for example, is widespread, and can be demonstrated as a surface component of many epithelia [6]. Our own data on epidermal differentiation shed a little light on this area. Murine epidermis expresses both syndecan-1 (predominantly) and syndecan-4. Single knockout of the corresponding genes does not impact epidermal morphology in the newborn or adult [35], but this is not the case for the double knockout. Here, the basal layers of the epidermis were disorganized, and subsequent analysis showed the abnormal expression of P-cadherin and also N-cadherin [35]. However, terminal differentiation in the strata granulosum and corneum appeared to be normal, so that the mice displayed no overt phenotype [35]. Altogether, the data suggest the possibility that syndecan-4 can replace syndecan-1 but it is only when the epidermis is syndecan-null that an observable phenotype is displayed.

## 3. Junctions and Syndecan-1

A key component of cellular morphology is the formation of junctions. Experimentally, syndecan-1 loss leads to a concomitant loss of E-cadherin in NMuMg cells, but whether this is partially or entirely due to transcriptional repression, trafficking alterations, or to cleavage and shedding of the cadherin is not clearly resolved. The connection between syndecans and cadherins has been made many times [36,37,38], though no data have yet shown a direct interaction between these two classes of cell surface receptor. It may well be that cadherins can be associated with syndecans in a complex with other receptors. [38]. NMuMg cells are particularly sensitive to the loss of syndecan-1. As typical epithelial cells, they express keratins as intermediate filament proteins [39], but whether they assemble substantial desmosomes or hemidesmosomes is uncertain; ultrastructural analysis of this cell line is sparse. In 3D cultures, these epithelial cells locate α6β4 integrin in a basal orientation by light microscopy immunocytochemistry, suggestive of hemidesmosome formation [40]. ZO-1 distribution also suggests tight junction assembly, but the formation of desmosomes is not documented. It may be that the lack of a substantial keratin/desmosome network facilitates the epithelial–mesenchymal morphological transition seen in this cell line.

## 4. Epithelial–Mesenchymal Transition (EMT) and Syndecan-1

**Figure 3:**
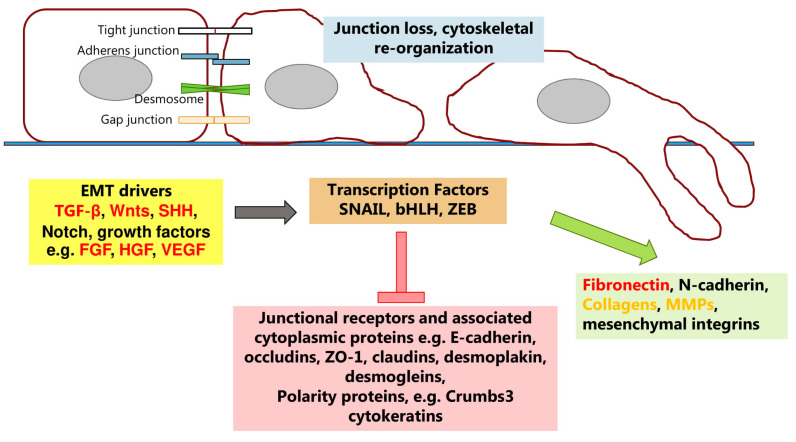
Schematic of some factors involved in epithelial–mesenchymal transformation (EMT). Polypeptides that promote EMT do so through transcriptional mediators that inhibit junctional and polarity protein expression but promote expression of proteins associated with a mesenchymal phenotype. Further detailed information can be found in [41,43,44,45,46,47]. Proteins that bind heparin/heparan sulfate are shown in red. Orange text denotes protein families where some members bind heparin or heparan sulfate.

There is now a wealth of literature regarding the process of epithelial–mesenchymal transition (EMT), with special emphasis on its relevance to tumor progression. Some of the key factors are shown in Figure 3. However, it is also now apparent that it is not a simple linear process. The concept has arisen that carcinomas, as they become invasive and break through the underlying basement membrane become mesenchymal. Dispersal through migration, potentially including the lymphatics or vasculature then leads to the establishment of tumor cells at distant sites. These then undergo the reverse (MET) process to form metastases. However, a recent review summarizes much data that are not easily reconciled to this simple paradigm [41]. Partial EMT is possible and many invasive carcinomas retain some epithelial molecular characteristics. For example, most invasive ductal carcinomas of the breast are E-cadherin positive, and the cadherin was shown to be a survival factor [42]. Here, collective cell invasion may be characteristic. An important set of criteria and guidelines for EMT (and MET), including in cancer, have been published recently [43].

The most well-known protagonist that promotes EMT is transforming growth factor-β (TGF-β), also known as a promoter of fibrosis in chronic inflammation [44,45,46]. In many epithelia it will promote loss of E-cadherin, with its replacement by N-cadherin which has lower affinity in cell–cell adhesion [47]. Key transcription factors include Snail, Slug and Zeb1/2. These trigger cadherin switching in addition to up-regulation of vimentin expression and ECM proteins such as fibronectin (Figure 3). From the many examples of this pathway it is safe to assume that TGF-β can promote EMT in the continued presence of syndecan-1. Indeed in NMuMg cells, TGF-β promotes chondroitin/dermatan sulfate substitution on syndecan-1 [48]. This suggests that the pathway that leads from syndecan-1 downregulation in vitro to a mesenchymal phenotype may not be a canonical EMT pathway. Indeed when considering epithelia in culture, vimentin and fibronectin as indicators of EMT may be unsafe, since many cell lines express these proteins as an adaptation to culture.

Our own preliminary data with NMuMg cells support the view that deletion of syndecan-1 does not initiate a classical EMT program; moreover, there are complex transcriptional changes. A possibility remains that other pathways than EMT are being implemented. It is known, for example, that alongside TGF-β, EMT can also be promoted by Wnt and Notch pathways (Figure 3). Many of these key molecules bind to heparan sulfate, e.g., TGF-β, Wnts, FGFs. Moreover, a further dimension to this puzzle may be cytosolic calcium, a key second messenger with impact on the actin cytoskeleton. We have reported that the epidermal phenotype in murine skin that is syndecan-null closely resembles that seen by deletion of the TRPC4 gene (transient receptor potential canonical 4). This stretch-activated calcium channel is present in epidermis and its deletion in epidermal cells leads to elevated cytosolic calcium, exactly as seen by deletion of syndecan-1 and -4 [35]. Other work has implicated TRPC6 and/or 7 as being subject to regulation by syndecan-4 in fibroblasts and kidney cells [35,49]. This regulation of TRP channels by syndecans appears to be an ancient property since we could also demonstrate it in *Caenorhabditis elegans* [35]. Calcium is known as an important player in determining cellular phenotype, notably junction formation, and can be a factor in EMT [50,51,52,53].

## 5. Regulation of Syndecan-1 Expression

Many reports have shown that syndecan-1 levels are altered in carcinomas of several types. The underlying causes of these changes are for the most part poorly understood. At the transcriptional level, the promoter of the human *SDC1* gene is not well characterized. It does contain a DR-1 element that is responsive to farnesoid X-receptor isoforms [54]. Of potential relevance to prostate carcinoma, the Zeb1 transcription factor, known to be a relevant factor in EMT, has been demonstrated to bind an E-box in the *SDC1* promoter and silence expression [55]. In the context of gynaecological tumors, estrogen receptor α signaling negatively regulates *SDC1* expression [56]. The murine *SDC1* promoter has been characterized and was shown to contain TATA and CAAT boxes, E-box, and binding sites for Sp1 and NF-kB [57]. Possibly the Sp1 sites represent a route to constitutive expression [58]. There is also a binding site for Wilms tumor suppressor 1 that leads to increased syndecan expression of potential relevance to kidney epithelial differentiation [59]. It has also been proposed that retinoid suppresses *SDC1* transcription in differentiating muscle cells, in a mechanism independent of E-box and FGF and TGF-β [60].

In many cancers, stromal expression of syndecan-1 is induced and often is an indicator of poor prognosis reviewed in [61]. This stromal source of the proteoglycan could be fibroblast, whose activation into cancer-associated fibroblasts has been described [62,63]. Blood vessels arising from tumor angiogenesis are a potential source, but in some cases, e.g., advanced breast cancer, some syndecan-1 is clearly associated with the collagenous extracellular matrix [64]. This is apparently derived from shedding at the cell surface of epithelial and/or stromal cells. Syndecans are exquisitely sensitive to a number of proteinases, notably MMPs, and there are many instances of upregulated MMP expression in tumor progression [65,66]. A “hot spot” for MMP cleavage of syndecan-1 (and other syndecans) lies in a membrane proximal region. Cleavage results in the release of a large portion of the core protein with glycosaminoglycan chains attached [67,68]. These can function as competitive inhibitors of the cell surface population, or may bind ligands and present them to cell surface receptors [6]. Since in many cases, the presence of stromal syndecan-1 is a sign of tumor aggressiveness, it appears likely that shed syndecan-1 is a mediator of invasion, proliferation and permissive alterations in the tumor microenvironment. In the breast cancer field, for example, shed syndecan-1 promotes invasive behavior, in a manner sensitive to the MMP inhibitor, TIMP1, and also triggers loss of E-cadherin [69].

Little is known regarding the regulation of syndecan-1 expression by mesenchymal cells, such as the cancer-associated fibroblast. Some fibroblasts in culture express this proteoglycan [35] and they can resemble “activated” fibroblasts, with pronounced microfilament bundles containing α-smooth muscle actin and OB-cadherin [70] at adherens junctions [36]. More than 20 years ago, Jalkanen’s group described an FGF-responsive enhancer region in the *SDC1* gene that was specifically activated in mesenchymal cells [71], but this has not been further investigated. However, an interesting more recent report relating to breast cancer showed that ionizing radiation triggered senescence in fibroblasts. This was accompanied by autocrine TGF-β activation and signaling, leading through Smads and Sp1 to elevated syndecan-1 expression [72]. Moreover, the triple-negative aggressively invasive MDA-MB-231 breast carcinoma line could also be a source of TGF-β for paracrine activation of syndecan-1 expression. By contrast, it appears that a number of epithelia are subject to post-translational upregulation of cell surface syndecan-1 by TGF-β [73]. In this case, the mechanism is protein kinase A-mediated, including a key cytoplasmic serine residue phosphorylation that led to elevated transport to the cell surface (ser286 at the C1/V boundary). This provides a clue with regard to the frequent observation that cytoplasmic syndecan-1 accumulates in many carcinomas (Table 1). There may be scope for the use of phospho-specific antibodies to examine this further.

An important adjunct to shedding is the enzyme heparanase. There are two isoforms in the human, but only heparanase-1 has enzymatic activity [6]. This enzyme is notably upregulated in a number of cancer types and is the focus of trials to determine if its inhibition can ameliorate tumor aggressiveness [74,75]. Heparanase selectively cleaves HS chains, liberating oligosaccharides that may be biologically active [76]. In addition, removal of HS by the enzyme exposes the syndecan core protein, which becomes even more sensitive to protease cleavage [77].

In 2019, an in-depth study of pancreatic ductal adenocarcinoma shed important light on another feature of syndecan localization [78]. Expression of oncogenic K-Ras led to upregulation of syndecan-1 on the surface of tumor cells, in a process mediated by MEK. The proteoglycan was then involved in macropinocytosis and was, moreover, required for tumor progression in this model. Roles for the G proteins Arf6 and Rac1 were described and interactions of the syndecan C2 domain with syntenin were also required for the endocytic process.

Involvement of syndecans in endocytic events has been highlighted previously. Hepatocytes are enriched in syndecan-1, where it performs essential roles in the clearance of specific lipids from the plasma [79]. All this highlights that although known as a cell surface HSPG, syndecan-1 can have cytoplasmic localization, and this is reported in several cancer studies (Table 1). In a much older study, for example, syndecan-1 had a lysosomal distribution in poorly differentiated breast carcinoma [80]. Altered subcellular distribution of syndecan-1 may be a significant facet of its biology, and there are also reports of its nuclear localization, with impact on transcription [81].

**Table 1:** Syndecan-1 (CD138) in Carcinomas.

Tissue	Normal Distribution	Carcinoma	Microenviroment	References
Skin	Viable layers of keratinocytes positive	Basal and squamous cell positive, decrease correlates with aggressiveness.	Stroma positivity correlates with aggressiveness	[82,83,84]
	Melanocytes negative, dermis negative	Malignant melanoma negative		
Oral cavity	Viable layers of epithelium positive Stroma negative	Oral quamous; decrease is adverse prognostic factor. Squamous head and neck; decreased levels correlate with poor prognosis	Stroma positivity relates to invasive activity.	[84,85,86,87,88,89]
Airway lung	Most epithelial cells positive, often low levels	SCLC, NSCLC; loss correlates with dedifferentiation and decreased survival.	High serum levels correlate with poor prognosis.	[90,91]
Breast	Low levels in ducts, myoepthelia and lobules	Elevated expression correlates with ER^-^ status, tumor grade, poor prognosis. Loss of membranous staining and acquisition of cytoplasmic staining equates with poor prognosis. May be a marker for triple negative inflammatory carcinoma.	Stromal staining, particularly in association with desmoplastic collagen is a poor prognostic indicator. Ectodomain in concert with αvβ3 integrin may be causal in collagen reorientation.	[64,92,93,94,95]
Ovary	Negative	Expression relates to tumor grade, often cytoplasmic and nuclear.	Stromal presence correlates with decreased survival.	[96,97]
Stomach	Parietal, chief, columnar and mucous-secretory cells positive	Low epithelial levels correlate with intestinal forms, depth of invasion, increased grade and tumor size	Stroma—as for epithelia	[98,99,100,101,102]
Colon	Most epithelial cells including crypt and goblet cells positive	High levels in adenoma, decreased in adenocarcinoma. Low levels associate with tumor recurrence, metastasis and poor survival	Positive tumor-associated fibroblasts relate to poorer prognosis	[103,104,105,106]
Liver	Dominant proteoglycan of hepatocytes, mostly basolateral	More uniform distribution, some cytoplasmic and nuclear staining. In hepatocellular carcinoma with no cirrhosis, reduced levels correlate with poor differentiation and metastasis. Elevated levels in HCC with cirrhosis.	Elevated serum levels correlate with tumor recurrence and decreased survival.	[107,108,109,110,111,112,113]
Pancreas	Low levels in ductal cells, less frequent in acinar cells, islets negative.	Pancreatic ductal adenocarcinoma, increased levels also in metastases.	Stromal staining correlates with worse progrnosis, independent of stage or grade.	[114,115,116]
Prostate	Epithelia positive- basal orientation.	Increased cytoplasmic expression associated with stage, Gleason grade and metastasis, and is a predictor of recurrence.	Positive stromal cells can be present in high grade tumors. High serum levels relate to worse prognosis.	[117,118,119,120,121]

Global reviews of syndecan-1 in tumors are references [61,122,123].

## 6. Syndecan-1 in Carcinomas

**Figure 4:**
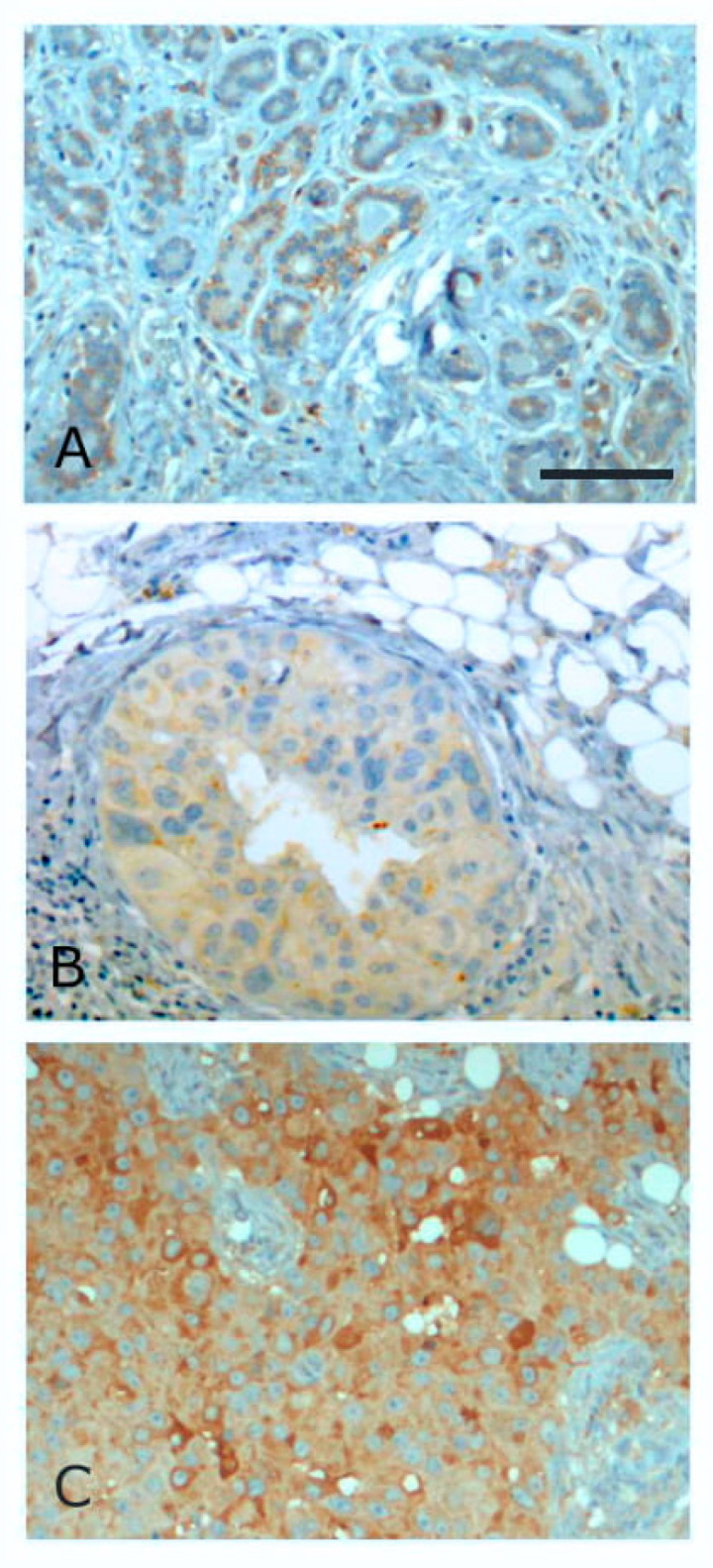
Immunoperoxidase staining for syndecan-1 in breast tissues. (**A**)—benign hyperplasia, (**B**)—ductal carcinoma in situ, (**C**)—infiltrating ductal carcinoma. In these carcinomas, there is loss of membrane staining but acquisition of general cytoplasmic staining. Scale bar = 100 µm.

Of the four syndecans in humans, syndecan-1 has by far received the most attention in the context of tumor progression. There are more reports on this proteoglycan than the other three combined. Syndecans and other cell surface HSPGs have attracted attention in the oncology field for several reasons. In the first place, many potent growth factors bind to HS chains and may concentrate them at the cell surface, where they can bind high-affinity receptors and trigger signaling. Syndecan-1, by virtue of its widespread presence and abundance in epithelia has then been of interest in a wide variety of carcinomas. Most of these studies are correlative, but nevertheless some clear parallels between syndecan-1 expression and prognosis have emerged. Table 1 lists some of the major tumor types and the potential involvement of syndecan-1. Figure 4 shows examples of syndecan-1 in breast carcinoma.

Some interesting overall observations can be made. In most normal epithelia, syndecan-1 is present, frequently with a basal or basolateral distribution. With oncogenic transformation, the amount of syndecan-1 and its distribution can change. Frequently, but certainly not uniformly, loss of syndecan-1 correlates with tumor aggression, grade, invasive behavior and poorer prognosis. In some gastrointestinal tumors, e.g., hepatocellular carcinoma and pancreatic ductal adenocarcinoma, levels of syndecan-1 can be increased. What becomes clear, however, is that beyond overall levels of the proteoglycan seen by immunohistochemistry, the fine localization is of great importance. Loss of cell surface staining, but acquisition of cytoplasmic or nuclear staining can be a sign of tumor progression and worse prognosis. This is particularly seen where stromal cells or the stromal matrix become positive. In virtually all tumor types, stromal syndecan-1 is an indicator of poor prognosis. It also appears that increased serum levels in patients is a similar sign of worse outcome. This would indicate that syndecan-1 shedding, presumably from the tumor and/or stroma has negative connotations.

## 7. Syndecan Roles in Murine Breast Cancer Models

Space precludes a comprehensive analysis of all mouse cancer models and involvement of syndecan-1. However, there are some insights into syndecan function that can be gleaned from some breast cancer model studies. In 2000, Alexander et al. [124] showed that the syndecan-1 null mouse was resistant to tumor formation induced by Wnt-1. However, a follow-up study [125] indicated that the major impact of syndecan-1 was not on Wnt signaling per se, but an alternate pathway that stabilized β-catenin/TCF-responsive tumor precursor cells. That pathway remains obscure. A third study [126] showed that there was a growth promoting loop between breast cancer cells and the stromal compartment, which was dependent on heparan sulfate-bearing syndecan-1. In these cases, it is interesting that the effects are syndecan-1-specific and there appears to be no compensatory role for other syndecan family members.

One further study sheds light on syndecan-extracellular matrix interactions. It is known that single or small clusters of breast carcinoma cells can reside in distant sites in a dormant or non-proliferative state. Weinberg’s group [127] have shown that there is an absence of β1 integrin-focal adhesion kinase signaling that could lead to proliferation. In its place, syndecan interactions with the matrix signalled a quiescent state. This was achieved through cytoplasmic domain interactions with a PAR1/PAR6γ/atypical protein kinase C complex. Specifically it was proposed that the C2 region of syndecans, including syndecan-1, could interact with PDZ domains intrinsic to PAR proteins [127]. In cases where cells were of s highly aggressive type, this pathway could be overcome. However, this study is unique in several respects. Interactions between the C-terminal EFYA motif of syndecan C2 regions and the polarity complex of PAR/aPKC has not been identified previously. Syndecan interactions with extracellular matrix are well known, and in fibroblasts promotes focal adhesion assembly [4,128]. Largely, however, these interactions also involve integrins, but in this specific case, syndecan-matrix interactions are independent. This presents an example where syndecan signaling can take place autonomously and promotes quiescence.

## 8. Targeting Syndecan-1 in Tumors

Syndecan-1 is highly expressed in multiple myeloma and is now considered as an important target [129]. A chimeric targeting antibody, indatuxumab ravtansine is under scrutiny [130,131]. The antibody component was developed from the well-known and widely used BB-4 antibody recognizing the core protein ectodomain. It is conjugated to a cytotoxic drug, in this case a maytansine derivative. The conjugate has been used in several preclinical studies and has been in phase I/IIa clinical trial in myeloma patients [132]. Early signs are encouraging and it may perhaps be utilized in a combination therapy approach [133,134]. More recently, a second promising monoclonal antibody has been developed, VIS832 recognizing a distinct epitope from BB-4. Maximal binding requires two non-contiguous regions of the syndecan-1 ectodomain [135].

Preclinical studies have been extended to triple-negative breast cancer [136]. Cell lines and a xenograft model were examined, and indatuximab ravtansine was reported to be efficacious alone or in combination with paclitaxel. A study with a human phage display antibody against syndecan has reported inhibition of tumor vasculature maturation in both melanoma and ovarian cancer models [137]. Here, the principle is to alter the tumor microenvironment, and this study reported that an association between syndecan-1 and vascular endothelial growth factor receptor2 (VEGFR2) was broken in the presence of the antibody. Linkage between syndecan-1 and this receptor together with integrin in a ternary complex has been implicated at early stages of angiogenesis [138]. Moreover, shed syndecan-1 ectodomain can promote VEGFR2 signaling, again in a complex with integrin [139]. Very recently, syndecan-4 was reported to be essential in pathological angiogenesis, where VE-cadherin internalization was induced by VEGF-A signaling, through VEGFR2 [38].

This last study highlights two important features of syndecan biology once again. The first is linkage to cadherins, where in this case syndecan-4 was shown to associate with VE-cadherin at junctions. Second, syndecan-mediated internalization comes to the fore. The propensity for syndecans to be involved in receptor internalization may be very favorable in clinical and preclinical settings, since endocytic events that take bound antibodies into the cell will also carry conjugated drugs. Cell killing may be enhanced as a result. However, it is also clear from the known distribution of syndecan-1 that besides being enriched in some tumors and tumor microenvironments, it is widespread in normal epithelia. While levels may be low, it is a reminder that targeting syndecan-1 may have unwanted side-effects.

## 9. Conclusions

Proteoglycans are under ever-increasing scrutiny as participants in tumor progression. The overwhelming majority of the data apply to syndecan-1, and it is important to recognize that while many studies are correlative, there are now direct data implicating syndecan-1 with tumor aggressiveness. This is particularly relevant to myeloma, but may also be pertinent to some carcinomas. In recognition of this fact, research has moved from solely basic to include preclinical and more recently clinical studies with monoclonal antibody-directed therapeutic approaches. Since syndecan-1 is expressed in many normal epithelia it will be interesting to see whether toxic payloads can be delivered to tumors without severe side-effects. These clinical studies are at an early stage, but it certainly highlights that understanding syndecan function can be relevant to human disease.

The heparan sulfate chains of syndecans have an ability to interact with a wide variety of polypeptides, including potent growth and migration promoters [15]. It remains unclear how ligands binding to heparan sulfate chains trigger signaling through the core protein. However, an important facet of syndecan function is that they can associate with other classes of receptors through which signaling occurs, e.g., integrins, fibroblast growth factor receptors (FGFRs), VEGFRs and TGF-βRs, Frizzleds, Robo/Slit [6,17,38,138,140] Additionally, syndecan functions increasingly appear to include internalization of ligand-receptor complexes. This may be useful when targeting syndecans for therapeutic purposes.

A survey of the many studies on syndecan-1 and carcinomas shows that mis-localization of the proteoglycan may be a key feature. Therefore, studies on syndecans in disease should take account of their detailed distributions. In many cases, abnormal cytoplasmic, nuclear and stromal populations of syndecan-1 have been recorded. The molecular basis for these observations is largely unknown and stand out as an important area for future research. A detailed understanding of syndecan-1 core protein signaling through its cytoplasmic domain also remains obscure, despite this member of the family receiving more attention than any other. Overall, it can be concluded that syndecans, as a small family of transmembrane proteoglycans with a long evolutionary history, have an important role in many diseases, including carcinomas. They therefore remain an area for future exploration at several levels, molecular, structural, genetic and pathological.
